# Liquid Biopsy in Neurological Diseases

**DOI:** 10.3390/cells12141911

**Published:** 2023-07-22

**Authors:** Sunny Malhotra, Mari Carmen Martín Miras, Agustín Pappolla, Xavier Montalban, Manuel Comabella

**Affiliations:** Multiple Sclerosis Center of Catalonia, Department of Neurology-Neuroimmunology, Vall d’Hebron University Hospital, 08035 Barcelona, Spain

**Keywords:** microRNA, liquid biopsy, cfDNA, neurological diseases

## Abstract

The most recent and non-invasive approach for studying early-stage biomarkers is liquid biopsy. This implies the extraction and analysis of non-solid biological tissues (serum, plasma, saliva, urine, and cerebrospinal fluid) without undergoing invasive procedures to determine disease prognosis. Liquid biopsy can be used for the screening of several components, such as extracellular vesicles, microRNAs, cell-free DNA, cell-free mitochondrial and nuclear DNA, circulating tumour cells, circulating tumour DNA, transfer RNA, and circular DNA or RNA derived from body fluids. Its application includes early disease diagnosis, the surveillance of disease activity, and treatment response monitoring, with growing evidence for validating this methodology in cancer, liver disease, and central nervous system (CNS) disorders. This review will provide an overview of mentioned liquid biopsy components, which could serve as valuable biomarkers for the evaluation of complex neurological conditions, including Alzheimer’s disease, Parkinson’s disease, amyotrophic lateral sclerosis, multiple sclerosis, epilepsy, stroke, traumatic brain injury, CNS tumours, and neuroinfectious diseases. Furthermore, this review highlights the future directions and potential limitations associated with liquid biopsy.

## 1. Introduction

Neurological diseases present a formidable challenge in terms of accurate and timely diagnosis. Traditional diagnostic methods often require invasive procedures that take time and may lack the sensitivity and specificity for early-stage detection. In this regard, a recent non-invasive approach has emerged as a promising avenue for studying early-stage biomarkers: liquid biopsy. This innovative methodology involves extracting and analysing non-solid biological tissues using less-invasive approaches, providing valuable insights into disease diagnosis and limiting potential harm to the patient. Further, it offers a versatile screening platform capable of rapidly assessing various components with potential as specific liquid biomarkers for diagnosing and monitoring complex neurological conditions. A few examples include the use of exosomal microRNA as a potential biomarker for the diagnosis of Parkinson’s [1] and the significance of circulating microRNA as a drug resistance biomarker, as seen in epilepsy cases [2]. However, the challenges and limitations of liquid biopsy are the need for more participants in current studies, variations in laboratory protocols, and the fact that many diseases share common features.

## 2. Liquid Biopsy: Definition and Applications

**Figure 1:**
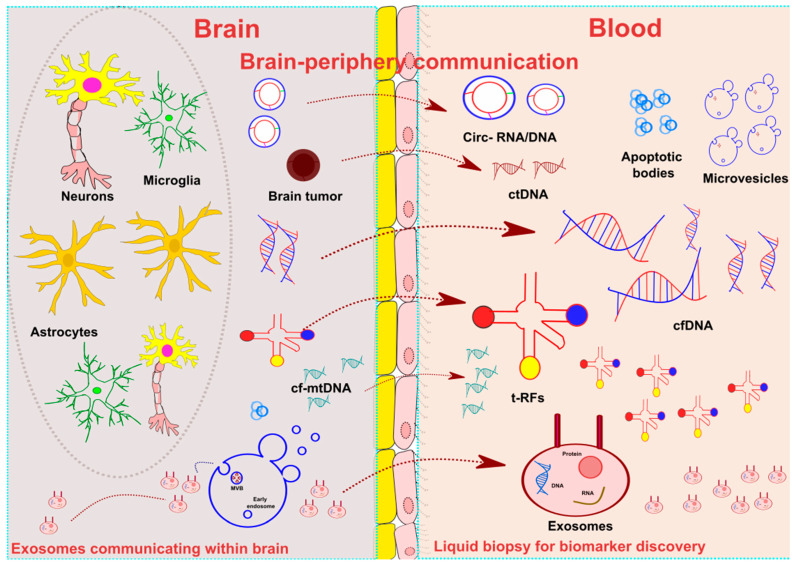
Overview of liquid biopsy. Figure showing brain–periphery communication. Central nervous system (CNS) cell populations, including neurons, astrocytes, and glial cells, release the principal elements of liquid biopsy components, including extracellular vesicles (micro-vesicles, exosomes, and apoptotic bodies), transfer-RNA (tRNA), circular RNA (circRNA), and cell-free (cfDNA) within the CNS. The most studied component of liquid biopsy constitutes exosomes. It contains functional microRNA (miRNA) and small RNA. A process of membrane vesicle trafficking performs the transfer of exosomes from the donor cell to the recipient cells. tRNA mainly comprises fewer than 90 nucleotides. tRNA-derived RNA fragments (t-RFs) act as essential mediators of CNS and immune communications via t-RFs. CircRNA is a single-stranded RNA, primarily present in circular form. Another component of liquid biopsy includes cell-free DNA (cfDNA), composed of double-helix DNA fragments released from CNS cells/nucleated cells and circulating freely in the bloodstream. Cell-free mitochondrial (cf-mtDNA) is a double-stranded fragment produced by dysfunctional mitochondria. Circulating tumour DNA (ctDNA) is produced by tumour cells; because of their half-life, these are real-time indicators of disease status in the body.

Liquid biopsy involves extracting and analysing non-solid biological tissues, such as serum, plasma, saliva, urine, and cerebrospinal fluid (CSF), without requiring invasive procedures. Liquid biopsy is considered the most recent, advanced, and non-invasive approach for discovering biomarkers related to disease prognosis and treatment response status. This technique consists of evaluating several components in the fluid compartments, which include extracellular vesicles (EVs) (exosomes, microvesicles, and apoptotic bodies), microRNA (miRNA), cell-free DNA (cfDNA), cell-free mtDNA (cf-mtDNA), cell-free nuclear DNA (cf-nuDNA), circulating tumour cells (CTCs), circulating tumour DNA (ctDNA), transfer RNA (tRNA), and circular DNA or RNA (circ-DNA/RNA). Figure 1 summarises the key components of liquid biopsy in neurological diseases. 

EVs are single-lipid membrane vesicles secreted by cells into all body fluids, which can be classified based on their size and origin into three major types. The most studied EVs are exosomes, whose average diameter ranges between 30 and 150 nm, and are the smallest vesicles. Exosomes are produced by the endosomal pathway and release extracellular components, which then participate in cell-to-cell communication and the transport of proteins, nucleic acid, and metabolites across cells. The second EV type is micro-vesicles (MVs), whose average size ranges between 100 and 1000 nm and are produced by blebbing of the plasma membrane. Lastly, apoptotic bodies, with an average size of 50 to 5000 nm, are produced by dying cells [3]. Cells from the central nervous system (CNS), such as neurons and glial cells, are known to release EVs into the extracellular space, where they can be identified by various methods, including specific cell surface markers or epigenetic information. Epigenetically, the methylation patterns of DNA within EVs can be studied in serum, plasma, or CSF samples, and their comparison with the already available atlas may lead to the identification of the cell types that produce EVs [4]. EVs have several functions in the body; in the CNS, they have been involved in various processes, including neurogenesis, neuron plasticity, and immune responses. Interestingly, it has been suggested that some EV cargoes modulate these critical CNS processes, which may lead to pathogenic degeneration when altered. As an example, the co-culture of adipose-derived mesenchymal stem cells (ADMSCs) or ADMSC-secreted EVs with neurons pre-exposed to oxygen–glucose deprivation (OGD) was associated with a significant reduction in neuronal cell death [5]. However, the effects of ADMSCs-derived EVs were reversed if they were pre-treated with an exosomal secretion inhibitor. This neuroprotective effect of ADMSCs-EVs was suggested to be mediated by miR-25-3p, insomuch as miR-25-3p oligonucleotide mimics reduced cell death [5]. 

Exosomes primarily contain miRNA and small RNA, with minimal linear RNA, which is the most common form of RNA in the cell nuclei. Exosomes act as bidirectional cargo in brain–peripheral communication and within the brain environment [6]. Additionally, exosome cargoes have been linked to the epigenetic regulation of neuroglial communication within the CNS and the brain–body axis [7]. The miRNA received by the recipient is called “exosome shuttle RNA”, which could potentially influence the latter’s production. One study suggested that tumour cells release proteins and exosomes under hypoxic conditions, promoting the tumoural microenvironment for angiogenesis and subsequent metastasis [8]. Likewise, microglia-derived exosomes have been shown to induce inflammatory responses in traumatic brain injury models [9,10].

miRNAs can also freely circulate in body fluids without being enclosed in exosomal cargoes. These have been studied in various neurological diseases, including stroke, where circulating serum miR-221-3p and miR-382-5p were significantly lower in patients with ischemic stroke than in healthy controls, highlighting the potential role of freely circulating miRNAs in diagnosing this condition [11]. 

Another component of liquid biopsy is cell-free DNA (cfDNA), which refers to the double-stranded DNA fragments released from nucleated cells that circulate freely in the bloodstream. Elevated levels of cfDNA have been associated with stroke severity and poor outcome during the first three months of recovery. Moreover, tumour-related cfDNA increases in plasma and serum, offering potential therapeutic targets for treating tumours based on their mutational profile and monitoring the treatment response [12]. cfDNA can be divided into two parts, namely cell-free mitochondrial DNA (cf-mtDNA) and cell-free nuclear DNA (cf-nu-DNA). cf-mtDNA consists of fragments from dysfunctional mitochondria that can be detected in the body’s fluid components. These fragments act as damage-associated molecular patterns (DAMPs), further triggering innate inflammatory responses. In the case of acute brain injury, elevated levels of cf-mtDNA were found in CSF and serum samples, and their levels were correlated with clinical severity and IL-6 cytokine response [13]. ctDNA refers to DNA released by tumour cells. In some cancers, higher levels of ctDNA have been linked to disease progression, with specific epigenetic profiles showing certain gene mutations [14].

Circulating tumour cells (CTCs) are the cells that get discarded from the primary site of tumours into the blood circulation. Understanding the metastasis cascade of CTCs is essential, which helps in identifying therapeutic targets against cancer metastasis. The low half-life of these cells makes them suitable for predicting the real-time situation of cancer in the body [15].

In recent years, tRNA-derived RNA fragments (t-RFs) have emerged as essential components of liquid biopsy due to their significant role in human diseases. Numerous studies have demonstrated that t-RFs function as homeostatic regulators of the post-stroke immune response, potentially serving as biomarkers for increased infection risk in stroke patients. The overexpression of t-RF-22-WE8SPOX52 has been shown to participate in the post-transcriptional regulation of genes by downregulating the Zbp1 protein, which acts as a DAMP sensor and induces NLRP3 inflammasome formation. Additionally, t-RFs mediate in post-damage communication between the CNSand immune cells [16]. Furthermore, a recent study involving the transfection of rat neuronal cultures with various t-RFs demonstrated neuronal swelling and subsequent cell death. This finding suggests that t-RFs mediate the molecular mechanism leading to neuronal cell death via glutamate-induced neuronal necrosis [17].

Circular RNAs (circRNAs) are RNA molecules that circulate freely in body fluids. Most circRNAs function as miRNA sponges or RNA-binding proteins [18]. CircRNAs have been implicated in various neurological diseases. Studies have indicated that circRS-7 acts as an miR-7 sponge in Alzheimer’s disease [19] and Parkinson’s disease [20], thereby modulating the expression of downstream genes such as cyclin B1 (G2/mitotic-specific) and CdK1 (cyclin-dependent kinase 1), which suppresses cell proliferation and cell migration [21]. 

A non-coding RNA (ncRNA) is an RNA molecule not translated into a protein. Long non-coding RNA (lncRNA) includes intergenic long intervening non-coding (lincRNA), intronic ncRNAs, and sense and antisense lncRNAs. lncRNAs have been implicated in several neurological diseases, including ischemic stroke. Few studies have shown that, in this entity, increased levels of non-coding nuclear abundant transcript 2 (NEAT2) were found in cultured brain microvascular endothelial cells (BMECs) after oxygen–glucose deprivation (OGD) and in isolated cerebral mice microvessels after middle cerebral artery occlusion (MCAO). Furthermore, the silencing of NEAT2 significantly worsens the OGD-induced expression of proapoptotic components, such as the Bcl-2 interacting mediator of cell death (Bim). This effect was studied in a NEAT2 knockout that presented a larger brain infarct size [22]. In a separate study, MCAO rats and OGD/R-treated neurocytes were investigated, and the up-regulated expression level of lncRNA maternally expressed 3 (MEG3) was observed. MEG3 and absent in melanoma 2 (AIM2) functioned as molecular sponges to suppress miR-485. AIM2 was further validated as a target of miR-485 [23]. In a study focused on patients with acute mountain sickness, the expression levels of ncRNAs were analysed. The results showed a significant up-regulation of lnc-CRKL-2 and lnc-NTRK3-4, and a down-regulation of RPS6KA2-AS1 and lnc-CALM1-7 [24]. 

## 3. Liquid Biopsy in Neurological Diseases

A wide range of techniques have been employed to study liquid biopsy in neurological diseases. These techniques include miRNA arrays for miRNA profiling, highly sensitive next-generation sequencing (NGS) for RNA sequencing, reduced representation bisulfite sequencing (RRBS), and Infinium MethylationEPIC BeadChip (EPIC) for analysing methylation patterns. Additionally, a highly sensitive proximity extension assay (PEA) was utilised, which combines an immunoassay for antibody recognition with highly sensitive qPCR. Another notable technique is small input liquid volume extracellular RNA (exRNA) sequencing (SILVER-seq), which enables the sequencing of this molecule in small liquid volumes. This section will provide the main findings of recent liquid biopsy studies in patients with neurological conditions. In addition, Table 1 summarises the main findings associated with selected biomarkers identified in liquid biopsy studies.

### 3.1. Alzheimer’s Disease

Alzheimer’s disease (AD) is the most common neurodegenerative disorder characterised by progressive cognitive decline and behavioural changes [44]. Key pathological hallmarks of AD include defects in amyloid precursor protein (APP) cleavage and beta-amyloid (Aβ) production, along with hyperphosphorylated tau protein aggregation, leading to an impaired synaptic function, conducive to neurodegeneration [45].

One approach utilises a highly prevalent DNA modification in the CNS, mainly to derive 5 hmC signatures from plasma cfDNA to segregate the multiple AD pathogenesis-related pathways in late-onset AD compared to healthy controls. Several genes were identified, such as *RABEP1, CPNE4*, *DNAJC15*, *REEP3*, *ROR1*, *CAMK1D*, and *RBFOX1*, showing a strong correlation with disease scores [46].

Components previously considered as cellular debris, such as EVs, circRNA, exRNA, and t-RFs, have been explored for their potential to serve as biomarkers in AD. For example, neuron-derived extracellular vesicles (NDEVs) were immunoprecipitated by targeting neuronal markers such as L1CAM from AD patients and healthy controls. Higher NDEV Aβ42 levels were associated with better scores in patients’ memory and cognitive status [25]. Another study using SILVER-seq technology observed differentially expressed candidate genes between AD patients and healthy controls, finding phosphoglycerate dehydrogenase *(PHGDH)* up-regulated in the AD brain. Increased plasma levels of *PHGDH’s* exRNA were associated with disease converters, reflecting the transition from normal cognition to impairment [26]. Additionally, a separate study reported increased cirHomer1 and hsa_circ_0131235 expressions associated with AD pathology [47,48]. The upregulation of t-RFs has also been reported in the hippocampus of AD patients, but the function of these small RNAs remains unclear [49,50]. Overall, these findings suggest that fluid biomarkers can be detected in the body fluids of AD patients using non-invasive techniques, serving as biomarkers for the early detection or progression of the disease. 

### 3.2. Parkinson’s Disease

Parkinson’s disease (PD), the second-most common neurodegenerative disease, is characterised by the loss of dopaminergic neurons in the substantia nigra and the widespread accumulation of Lewy bodies, mainly composed of fibrillar aggregates of the presynaptic protein α-synuclein [51]. Despite recent advances in diagnosing PD, including advanced imaging techniques, definite diagnosis remains limited to clinical observations, warranting a high need for reliable biomarkers to identify the early stages of the disease [52]. In this sense, the isolation of EVs from blood or other body fluids has emerged as a promising approach for diagnosing and monitoring PD progression.

Studies have shown that levels of α-synuclein inside plasma EVs are significantly greater in patients at early stages of PD than in healthy controls, indicating their potential as a diagnostic and prognostic biomarker [53]. Moreover, the identification and profiling of CSF and blood-derived miRNAs can aid in differentiating PD cases from healthy individuals. Several differentially expressed miRNAs have been identified, including miR-132-5p, miR-34c-3p, miR-132-3p, miR-19b-3p, miR-29c, miR-133b, and miR-331-5p [27,54]. Furthermore, EVs derived from plasma samples of PD patients showed decreased levels of hsa-miR-15b-5p, hsa-miR-138-5p, hsa-miR-338-3p, hsa-miR-106b-3p, and hsa-miR-431-5p, while levels of hsa-miR-30c-2-3p were increased. The expression of the genes hsa-miR-15b-5p, hsa-miR-30c-2-3p, hsa-miR-138-5p, and hsa-miR-338-3p was increased in the dopaminergic synapse and the PD pathway, indicating their potential role in modulating dopamine expression and their use as potential diagnostic biomarkers [28]. 

Saliva is another potential source of biomarkers for the early screening of PD. Commonly detected biomarkers in saliva include α-synuclein, protein deglycase (DJ-1), and oxidative stress markers such as hemeoxygenase-1 (HO-1), acetylcholinesterase (AChE), and total protein [55].

Although urine samples are highly diluted, they can also serve as a source of biomarkers for PD. Higher urine kynurenine (KYN) levels have been associated with early stages of PD and correlated with mild cognitive impairment and disease severity [56]. Additionally, cf-mtDNA has been identified as a potential biomarker, as its levels were significantly reduced in the CSF of PD patients compared to control diseases [57]. 

In summary, several approaches have been tested in various body fluids to diagnose PD earlier. These studies resulted in the preliminary identification of biomarkers for the disease, although further validation in larger cohorts is needed. 

### 3.3. Amyotrophic Lateral Sclerosis

Amyotrophic lateral sclerosis (ALS) is a fatal adult-onset neurodegenerative disorder characterised by the progressive loss of upper and lower motor neurons [58]. Although most ALS cases are sporadic (sALS), approximately 5–10% correspond to familial forms with mutations in diverse genes, including Cu^2+^/Zn^2+^ superoxide dismutase *(SOD1)* and TAR DNA-binding protein 43 *(TDP-43)*, among others [59]. Neuroinflammatory responses associated with the activation of microglia and astrocytes are prominent features of ALS and are believed to contribute to disease progression [60].

Recently, liquid biopsy approaches using CSF have been employed to identify potential biomarkers of ALS. The ultrasensitive protein detection method, PEA, was applied to CSF-EV samples, and it was found that the junctional adhesion molecule A (JAM-A) protein, chitinase-1, and tumour necrosis factor receptor-2 (TNF-R2) were significantly up-regulated in ALS cases. At the same time, myoglobin was down-regulated [61]. NGS was performed on exosome messenger RNA (mRNA) isolated from CSF. This methodology identified CUE domain-containing 2 *(CUEDC2)* as the top candidate biomarker for this disease, as it was highly increased in CSF from ALS cases [29]. Similarly, differential methylation markers were obtained from cfDNA isolation from CSF and plasma of ALS patients for biomarker discovery. This study identified a novel differentially methylated region in the rhomboid 5 homolog 2 *(RHBDF2)* gene in ALS patients compared to controls, highlighting its potential as an epigenetic biomarker of neurodegeneration [62].

A recent study identified a biomarker signature of ncRNA in serum samples of ALS patients, including hsa-miR-16-5p, hsa-miR-21-5p, hsa-miR-92a-3p, hsa-piR-33151, TRV-AAC4-1.1, and TRA-AGC6-1.1. This combination accurately differentiated ALS from non-ALS cases [30]. Furthermore, other studies have identified hsa-miR-4299 and hsa-miR-4649-5p as down-regulated and up-regulated miRNAs, respectively, in the plasma of sALS patients. Additionally, hsa-miR-663b and hsa-miR-4258 were significantly down-regulated in the CSF of patients, while no miRNAs were found to be up-regulated [31]. 

In summary, these liquid biopsy approaches offer promising insights into identifying potential biomarkers for ALS. However, further research is needed to validate and translate these findings into clinical practice.

### 3.4. Multiple Sclerosis

Multiple sclerosis (MS) is a disease that presents with neuroinflammation and neurodegeneration [63]. Several studies have searched for biomarkers in different clinical phenotypes of MS. Firstly, the presence of anti-myelin lipid-specific oligoclonal IgM bands (LS-OCMBs) in CSF is considered to be a predictor of the aggressive evolution of the disease. However, obtaining CSF through a lumbar puncture is an invasive approach. To overcome this issue, Iparraguirre et al. (2020) used less invasive approaches, i.e., peripheral blood mononuclear cells (PBMCs). Transcriptomic analysis was performed on these samples, and further bioinformatics analysis suggested that several free circular and linear RNA biomarkers were differentially expressed. After performing validation experiments using qPCR, only two circRNAs (hsa_circ_0000478 and hsa_circ_0116639) and two linear RNAs (*IRF5 and MTRNR2L8*) were found to be significantly differentially expressed between LS-OCMBS-positive and negative patients [64].

Exosomes derived from body fluids (serum, plasma, or CSF) of MS patients and healthy controls have also been exploited in various liquid biopsy approaches. One group reported an increased expression of Epstein–Barr virus (EBV) nuclear antigen EBNA1, and latent membrane proteins LMP1 and 2A on serum exosomes derived from patients with active relapsing-remitting MS (RRMS) compared to healthy controls or stable RRMS [65]. Another study demonstrated a diminished expression of hsa-miR-122-5p, hsa-miR-196b-5p, hsa-miR-301a-3p, and hsa-miR-532-5p in serum samples of RRMS patients compared to healthy controls. Interestingly, these miRNAs have been involved in direct cell-to-cell communication [33]. Lastly, exosomes were isolated from urine, plasma, and CSF samples from MS patients and healthy controls and the authors reported increased levels of miR-155-5p on day 6 before disease onset [34].

Two viruses are related to MS and demyelinating events pathogenesis. EBV is considered to be a causative event that can lead to MS development and could also be identified by detecting EBV-derived cell-free DNA in CSF or plasma samples [32]. Another viral pathogen related to demyelinating diseases is the human John Cunningham virus (JCV), causing progressive multifocal leukoencephalopathy (PML) [66], which mainly affects MS patients due to the reactivation of a latent JCV infection in the brain as a severe adverse effect originating from the immunosuppressive treatment [67]. The viral DNA can be detected in CSF by PCR assays, helping to arrive at an accurate diagnosis in combination with typical brain MRI findings [68].

These results suggest that MS patients showed a differential expression of several components in various body fluids, such as miRNA isolated from exosomes and several free-circulating linear and circular RNA biomarkers.

### 3.5. Epilepsy

Epilepsy is one of the most common neurological diseases [69], affecting over 70 million people worldwide [70]. It is a chronic disease that primarily affects the brain cortex as a consequence (or not) of CNS insults, predisposing patients to suffer seizures [69]. Therefore, patients with this condition may experience severe long-term consequences, including increased comorbidities, disability, and mortality [71].

Regarding disease epidemiology and pathogenesis, quantifying lncRNA in the peripheral blood of epileptic patients showed that molecules such as HOXA-AS2 and SPRY4-IT1 have a significantly increased expression in male epilepsy patients, suggesting that these two lncRNAs could potentially play a role in the pathogenesis of the disease [72]. In line with this, studying cfDNA methylation in serum or plasma samples could provide new biomarkers for identifying various stages of epileptogenesis and high-risk patients. This is supported by the fact that some neurodegenerative disorders involve methylation remodelling in the DNA of genes related to inflammation and disease duration, as observed in the anterior temporal neocortex and hippocampus of mesial temporal lobe epilepsy with hippocampal sclerosis (mTLE-HS) patients. Some of these methylation patterns may also be present in the circulation and derive from damaged neurons or glia [73]. For example, a study suggested that the mean baseline concentration of cfDNA was lower in patients with extratemporal lobe epilepsy (XTLE) than in control subjects. Furthermore, the maximum concentration of cfDNA after baseline measurement was significantly lower in patients with a disease duration of more than 18 years than in those with a disease duration of less than 18 years [74].

The most extensively studied liquid biopsy markers for epilepsy are free-circulating or EV-derived miRNAs originating from neurons or glia that circulate in the blood or CSF. In patients with temporal lobe epilepsy (TLE)—which comprises 30% of all epilepsy cases—and status epilepticus (SE), the EVs derived from CSF have been found to contain numerous differentially expressed miRNAs. TLE-derived EVs showed increased levels of miR-19b-3p and decreased levels of seven different miRNAs, while SE-derived EVs had increased levels of miR-21-5p, miR-451a, and six other miRNAs, along with a decreased expression of miR-204-5p [75]. Further, serum samples of TLE patients showed elevated levels of the lncRNA ILF3-AS1 compared to controls. This molecule is closely associated with epilepsy due to its role in promoting the expression of metalloproteinases (MMPs) and other pro-inflammatory cytokines commonly found in this disease [76]. Additionally, in mTLE-HS, six miRNAs were found to be differentially expressed compared to healthy controls. Only miR-8071 exhibited high sensitivity and specificity, and was additionally associated with seizure severity [35].

In other focal epilepsies, plasma samples are a good source of t-RFs. Specifically, 5′GluCTC-t-RF, 5′GlyGCC-t-RF, and 5′AlaTGC-t-RF are increased in samples prior to seizure compared to post-seizure samples, reflecting the potential role of t-RFs as non-invasive predictors of epilepsy risk [36]. Further, differences in the expression levels of microRNAs were observed between epileptogenic and non-epileptogenic tuberous sclerosis complex (TSC) tubers. Epileptogenic tubers showed a significant increase in miR-142-3p, miR-223-3p, and miR-21-5p compared to non-epileptogenic TSC tubers. These miRNAs are known to activate toll-like receptors 7 and 8 (TLR-7/8), resulting in the propagation of neuroinflammatory responses [77]. Moreover, neuronal cfDNA found in plasma could help to identify somatic epilepsy mutations, as this cfDNA could have crossed the BBB due to its increased permeability derived from brain damage [78]. 

Regarding treatment, a few biomarkers have been linked to drug resistance or favourable surgical outcomes. miR-301a-3p was significantly reduced in the serum of patients who developed drug resistance in cases of refractory epilepsy (RE). Researchers concluded that miR-301a-3p could serve as a potential biomarker not only for mesial temporal lobe epilepsy but also for predicting sudden death. Likewise, miR-654-3p may help in predicting favourable surgical outcomes in mTLE-HS patients [36].

Altogether, several studies have outlined the role of exosome-derived miRNA, cfRNA, cfDNA, and tRNA in the early detection of epilepsy, seizure severity, and drug resistance. These studies are in the exploratory stages; until now, no biomarkers have been used in the clinical setting. 

### 3.6. Stroke

Stroke is the second leading cause of death and a significant cause of disability worldwide [79]. There are two major types of strokes: acute ischemic stroke (AIS), which accounts for approximately 80% of cases and is caused by the interruption of cerebral blood flow, and haemorrhagic stroke, which represents the remaining 20% and is the consequence of a ruptured blood vessel [80].

Several studies have investigated the role of circRNAs in this entity. A study showed that circSCMH1 levels are diminished in AIS. Additionally, functional recovery was promoted in rodent and non-human primates by using rabies virus glycoprotein-circSCMH1-extracellular vesicles [81]. A separate study found that the expression levels of circTLK1 were significantly increased in brain tissue and plasma isolated from animal models of AIS. Moreover, it was observed that it improved long-term neurological deficits and significant reductions in infarct volumes and neuronal injury after knocking down circTLK1 in an animal model of AIS [82]. Furthermore, it was found that circCDC14A was significantly increased in lymphocytes and granulocytes of AIS patients [83]. In another study, the expression levels of circRNA_0001599 were found to be positively correlated with the National Institute of Health´s Stroke Scale Score (NIHSS) and infarct volumes [84].

These studies suggest that circRNAs can be used as biomarkers and therapeutic targets in AIS.

### 3.7. Traumatic Brain Injury

Traumatic brain injury (TBI) is one of the leading causes of long-term disability. It is characterised by an immediate primary mechanical injury followed by a secondary injury that can persist over time due to pathophysiological changes [85,86]. TBI can be classified into mild (mTBI) or severe (sTBI), urging molecular approaches to evaluate this entity in the emergency setting.

A recent study investigated cfDNA levels in patients with TBI. The total amounts of cf-mtDNA (mtCOXIII, mtNADI) and cf-nuDNA (nuACTB and nuSIRT1) were analysed using qPCR from serum samples of healthy controls and patients with mTBI and sTBI. Significantly higher levels of all four cfDNA markers were observed in patients with severe TBI (sTBI) compared to healthy controls [37]. In another study, attenuated total reflectance (ATR)–Fourier transform infrared (FTIR) spectroscopy was used to analyse serum samples from mTBI patients and healthy controls. Machine learning algorithms were then developed using the data obtained to identify which patients with mTBI were most likely to present a positive CT scan. This approach presented a very high sensitivity and specificity, distinguishing patients with mTBI from controls [87].

As aforementioned, most of the work has been focused on searching for biomarkers for differentiating mTBI from sTBI by using several components of liquid biopsy, particularly cfDNA and cf-mtDNAs. The applicability of these selected biomarkers could be used in clinical settings as they present a high sensitivity and specificity, although further validations are needed. 

### 3.8. CNS Tumours

Primary CNS tumours are highly heterogeneous and can be benign or malignant. Gliomas are the most frequently diagnosed among the latter, and glioblastoma multiforme (GBM) is the most aggressive form, and is particularly prevalent in adults [88]. These tumours are challenging to treat, with an overall 5-year survival rate of 35% [89], dropping to 5% for GBM [39]. Accordingly, a significant amount of research in liquid biopsy has been focused on GBM, including EVs, cell-free DNA, RNA, and CTCs. 

EVs isolated from blood and CSF samples revealed that isocitrate dehydrogenase 1 (IDH1) mutation, IDH1^R132H^ is a diagnostic biomarker of GBM. Further analysis revealed that higher concentrations of EVs containing this molecule are related to tumour recurrence in patients who underwent resection [38].

Liquid biopsy from CSF samples revealed that combinations of miRNAs, such as miR-2 and miR-15b, could distinguish GBM from primary CNS lymphoma, with a high sensitivity and specificity. Furthermore, a combination of a signature of nine miRNAs was found to correlate with a higher sensitivity and specificity to tumour volume [39]. Additionally, a panel of exosomal miRNAs could serve as predictive biomarkers for GBM patients, aiding in monitoring the treatment response to chemotherapy and drug resistance [38].

ctDNA of some tumour suppressors, such as P53 and PTEN, can be detected in all recurrent gliomas, making ctDNA a valuable biomarker for predicting the response to adjuvant therapies, providing real-time information about tumour progression and recurrence. In the same study, the authors found that mutations in ctDNA of RB1 and EGFR could be used as biomarkers for predicting the IDH-wildtype subtype of GBM and, consequently, the malignancy of the tumour [40].

Overall, several approaches have suggested a promising role of EVs, CTCs, and ctDNA in determining tumour progression, treatment effect, early diagnosis, and tumour recurrence. 

### 3.9. Neuroinfectious Diseases 

Neuroinfectious diseases are pathological conditions that produce brain or spinal cord inflammation triggered by pathogenic microorganisms such as bacteria, viruses, parasites, and fungi. Current diagnostic methods rely on a microbial examination of biological samples, particularly CSF. Examples include microscopic observation, Gram staining, cultures, pathogen-specific antibody reactions, and the detection of pathogen nucleic acids using PCR, among others. However, these diagnostic procedures have some limitations, as some lack sensitivity and require time to arrive at a final diagnosis. As a result, there is an urgent need to find more accurate, fast, and comprehensive techniques to accurately diagnose such diseases [90].

For bacterial pathogens, such as *M. tuberculosis,* the amplification of the cfDNA IS6110 sequence in the CSF of tuberculosis meningitis patients is more sensitive than some traditional diagnostic protocols. NGS cfDNA can be applied to other infections, such as *C. canimorsus meningitis*. Moreover, antibiotic exposure does not affect this diagnostic method [41].

Regarding viral CNS infections, determining cf-mtDNA levels in HIV-infected patients, with or without highly active antiretroviral therapy (HAART), may reflect the current status of CNS damage. In this sense, an inverse correlation between the levels of cf-mtDNA in CSF samples and proteins necessary for angiogenesis and iron metabolism was observed, suggesting that these molecular events are related to CNS damage [32].

Parasitic infections caused by *T. solium*, a causative agent of neurocysticercosis, can be detected in CSF and urine samples via cfDNA determination by amplifying the pTsol9-gene through PCR primers [42]. The same approach can be applied to *P. falciparum*, which causes cerebral malaria, by measuring total plasmatic cfDNA: both host cfDNA in response to the infection and parasite cfDNA [43]. 

Finally, NGS can be useful for pathogenic agent detection when performed on CSF samples in presumed or definitive cases of neuroinfectious diseases. Bacterial capture sequencing (BacCapSeq) and all vertebrate virus sequencing (VirCapSeq-VERT) show promising capacities for detecting various pathogens. However, the clinical application of these techniques still needs to be explored [91] (Table 2).

## 4. Conclusions and Future Directions

Liquid biopsy assesses components that are shed from cells into body fluids, and, over the last decade, several sensitive technologies have emerged for detecting them. Previously considered as insignificant or waste material, these technologies have elucidated their functional roles in disease progression or as prognostic biomarkers. Liquid biopsy represents a sensitive and non-invasive approach for early diagnosis, the identification of therapeutic targets, real-time disease surveillance, and the monitoring of treatment effects. Furthermore, this technique has already been employed as an outcome measure in clinical trials, mostly in CNS tumours. However, further investigation is needed for other CNS conditions. 

Despite the promising potential of non-invasive approaches in diagnosing, prognosis, and monitoring neurodegenerative diseases, the clinical application of these biomarkers still needs to be improved when compared to traditional diagnostic techniques, it being essential to acknowledge the several inconveniences that it faces. The number of participants in liquid biopsy studies is often insufficient, there is heterogeneity among participants, and many neurological diseases share common features often accompanied by neurodegeneration/neuronal damage. Consequently, many biomarkers encountered in liquid samples result in overlapping conclusions. 

The future of liquid biopsy will depend on the identification and validation of genetic, epigenetic, and protein components through the standardisation of protocols in larger multicentric cohorts. Additionally, it will be crucial to develop simplified tests that facilitate a seamless transition from the laboratory to clinical settings, ensuring cost-effectiveness and reducing the expertise required for implementation. This approach will facilitate the integration of liquid biopsies into the existing extensive clinical and radiological evaluations, making the process more accessible and efficient.

## Figures and Tables

**Table 1:** Selected liquid biopsy biomarkers in neurological diseases.

Biomarker	Source	Main Findings
Alzheimer’s Disease		
*↑* Aβ42	NDEVs	- Associated with better memory and cognitive status of AD patients [25]
*PHGDH’s* exRNA	Plasma	- Associated with disease converters, transition from normal to cognitive impairment [26]
Parkinson’s Disease		
*↓* miR-19b, miR-29c and miR-133b *↑* miR-132 and miR-331-5p	Blood, CSF, and exosomes	- Their target genes are associated with the disease: *FMR1*, *LRRK2*, *COQ2*, *HIP1R*, *ATP13A2*, *SYT11*, *RAB39B*, *CHCHD2*, *PLA6G2*, *EDN1*, and *SNCA* [27]
*↑* hsa-miR-30c-2-3p *↓* hsa-miR-15b-5p, hsa-miR-138-5p, hsa-miR-106b-3p	Plasma-EV	- Potential biomarker for PD diagnosis. Diagnostic accuracy increases when combined [28]
Biomarker	Source	Main Findings
Amyotrophic lateral sclerosis		
CUEDC2 exosomal mRNA	CSF EVs	- Highly increased in CSF from ALS cases [29]
hsa-miR-16-5p, hsa-piR-33151 and TRV-AAC4-1.1	Serum	- Significantly altered expression in ALS cases. Exhibited high sensitivity and specificity when classifying ALS and non-ALS cases [30]
*↓* hsa-miR-4299 *↑* hsa-miR-4649-5p	Plasma	- Differentially expressed in sporadic ALS cases [31]
*↓* hsa-miR-663b and has-miR-4258	CSF	- Downregulated in sporadic ALS cases [31]
Multiple sclerosis		
EBV-derived cfDNA	CSF/plasma	- It allows for further investigation on MS etiology and comparison of changes in biomarker levels with neuroimaging biomarkers and clinical status of patients [32]
*↓* hsa-miR-196b-5p, hsa-miR-532-5p, hsa-miR-122-5p, and hsa-miR-301a-3p	Serum	- Downregulated during relapse in RRMS. All miRNAs are involved in direct cell-to-cell communication [33]
*↑* miR-155-5p	CSF/plasma/urine exosomes	- Expression increases before MS onset [34]
Epilepsy		
miR-8071	Plasma exosomes	- Associated to severity of seizures [35]
miR-654-3p	Blood	- Predicts good surgical outcomes [36]
Stroke		
circRNA_0001599	Plasma	- Positively correlated with the National Institute - of Health Stroke Scale Score and infarct volumes [18]
Traumatic brain injury (TBI)		
*↑* ccfDNA: mtDNA (mtCOXIII, mtNADI) and nuDNA (nuACTB, nuSIRT1)	Serum	- Significantly high levels in sTBI compared to - healthy controls [37]
Biomarker	Source	Main Findings
CNS tumours		
IDH1^R132H^	Blood/CSF EVs	- Higher concentrations of EVs correlated to tumour recurrence in patients that underwent resection. Potential biomarker of early diagnosis of GBM [38]
miR-2 and miR-15b	CSF	- GBM survival rate is 5% [39]. miRNAs could distinguish GBM from primary CNS lymphoma with higher sensitivity and specificity [40]
Tumour suppressor genes-derived ctDNA (TP53 and PTEN).	Blood	- Detected in all recurrent gliomas. Provide real-time information of tumour progression and recurrence [40]
Mutation in RB1 and EGFR ctDNA		- Biomarker for predicting IDH-wild type subtype of GBM and, consequently, malignancy of tumour [40]
Neuroinfectious diseases Bacterial meningitis		
*Mycobacterium tuberculosis* cfDNA (IS6110 sequence)	CSF	- Its amplification is more sensitive than some traditional diagnostic protocols [41]
*Capnocytophaga canimorsus meningitis* cfDNA	Blood	- Diagnostic method is not affected by antibiotic exposure [41]
Neurocysticercosis (infection by *Taenia solium*)		
cfDNA: pTsol19-gene amplification	Urine/CSF	- Allows parasite detection, with subsequent amplicon sequencing to confirm positivity [42]
Cerebral malaria (infection by *Plasmodium falciparum*)		
Total cfDNA: host cfDNA (in response to infection) and parasite cfDNA	Plasma	- Biomarker of severity and, consequently, allowing for patients stratification by risk [43]

Aβ42 = amyloid-β 42; AD = Alzheimer disease; ALS = amyotrophic lateral sclerosis; cfDNA = cell-free DNA; CNS = central nervous system; CSF = cerebrospinal fluid; EBV = Epstein–Barr virus; EVs = extracellular vesicles; exRNA = extracellular RNA; GBM = glioblastoma; IDH = isocitrate dehydrogenase; miR = microRNA; MS = multiple sclerosis; PD = Parkinson disease; RRMS = relapsing–remitting multiple sclerosis; TBI = traumatic brain injury; NDEVs = neuron-derived extracellular vesicles; *↑* = upregulated; *↓* = downregulated.

**Table 2:** Primary limitations and possible solutions in liquid biopsy.

Primary Limitations	Possible Solutions
Small sample size	-Multi-centric samples collections
Heterogeneity in protocols	Expert consensus on the protocols
Heterogeneity of the participants	Correct clinical classification should be validated by traditional techniques and genetic background should be taken into consideration while conducting analysis
Expensive cost	Major laboratories should work on cost-effectiveness to get the easy penetration of the liquid biopsy test world-wide

## Data Availability

Not applicable.
